# Impact of Degradable Linkages on the Crystallization Behaviors of Polyethylene Mimics

**DOI:** 10.1021/acs.macromol.5c02706

**Published:** 2025-11-21

**Authors:** Jin Qian, Xiaomeng Li, Chuanbing Tang, Zhe Qiang

**Affiliations:** † School of Polymer Science and Engineering, 2629 The University of Southern Mississippi, 118 College Drive, Hattiesburg, Mississippi 39406, United States; ‡ Department of Chemistry and Biochemistry, University of South Carolina, Columbia, South Carolina 29208, United States

## Abstract

Incorporating degradable linkages into polyolefin backbones offers a promising route toward sustainable alternatives. For semicrystalline degradable polyolefins, understanding the impact of such linkages on their crystallization behaviors is important, since the crystallization process governs their morphology formation and thus dictates mechanical and thermal performance. Here, we studied the crystallization behaviors of degradable polyethylene (PE) mimics containing sparsely inserted ester linkages along the polymer backbone, which exhibit comparable mechanical and thermal properties to conventional PE. Two systems, HDPE-DM and LLDPE-DM, were investigated and compared with their commercial, non-degradable counterparts under isothermal and non-isothermal conditions. We found that ester incorporation does not alter the thermodynamics of the perfect crystal. However, relative to the non-degradable PE mimics (HDPE-M), the presence of ester linkages accelerates crystallization kinetics by reducing the overall crystallization activation energy and promoting chain folding. The crystallization activation energy of HDPE-DM was comparable to that of commercial HDPE, indicating that ester linkages can be introduced in polymer backbones without significantly impacting their crystallization behaviors and thus relevant industrial manufacturing processes. These findings provide new insights into how degradable linkages influence crystallization in degradable PE materials, which may inform the design and process of sustainable polyolefin materials with a balanced performance and recyclability.

## Graphical abstract

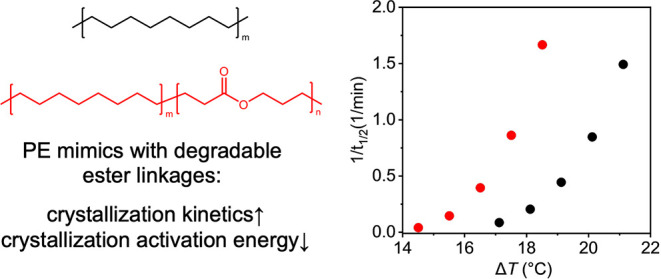

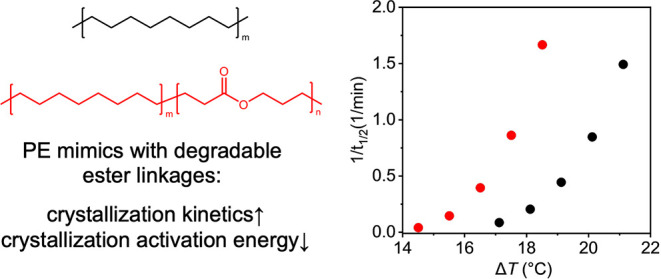

## Introduction

Polyolefins are among the most widely used polymer materials in the world, with applications spanning packaging, electronics, textiles, and automotive, making them integral to modern life.^1^ In particular, semicrystalline polyolefins such as polyethylene (PE) and polypropylene (PP) dominate global markets due to their satisfactory mechanical properties and excellent chemical resistance, which arise from their semicrystalline microstructures.^2^ Understanding and controlling the degree of crystallinity and crystalline morphology of polyolefins are therefore critical research areas, as they directly determine their material performance. Decades of research have shown that polymer structure and topology strongly influence the crystallization kinetics of polyolefins.^3–5^ For instance, short- and long-chain branching in PE disrupts chain packing, leading to thinner, more defective lamellae and slower nucleation and growth compared to linear PE.^6–9^ Similarly, copolymers or blends containing segments of different chemical identities often show crystallization behaviors distinct from their homopolymer counterparts, depending on the miscibility and compositions of the secondary segments.^3,4,10–14^ Parameters of industrial processing conditions such as cooling rate and applied shear can be employed to further modulate polyolefin crystalline morphology, enabling control of macroscopic properties and product performance.^15^

The chemically inert C–C backbone that imparts durability and chemical resistance to PE and PP results in their poor degradability, making them major contributors to persistent plastic waste in the environment. In recent years, significant efforts have been devoted to addressing the sustainability challenges of plastics, including the development of improved strategies for managing existing plastic waste,^16–22^ as well as the design of next-generation polymers with end-of-life considerations.^23–25^ A key effort in sustainable polymer development is controlling their semicrystalline morphologies to match the mechanical and thermal performance of conventional polyolefins, while imparting degradability in the polymer backbone for chemical recycling.^26–33^ One promising approach is to incorporate cleavable groups into the polyolefin backbone.^31,34–38^ Despite emerging research efforts in chemistry and materials development, the impact of degradable linkages on polyolefin crystallization behavior still remains to be further understood.^30,39–43^ The presence of functional linkages can influence chain packing, nucleation, and lamellar thickness, depending on linkage type and spacing. For example, Haider et al. showed that PE-like alternatives remain semicrystalline but exhibit reduced melting temperature and crystallinity relative to linear PE, suggesting that in-chain degradable linkages could disrupt chain packing.^39^ Likewise, Jang et al. demonstrated that higher ester contents progressively depressed melting temperatures (*T*_m_) and crystallinity of the linear PE-ester copolymer. However, when the ester content was lower than 3 esters per 1000 carbons, PE-ester preserved similar crystallinity as conventional PE, which suggested a spacing-dependent effect on lamellar assembly.^35^ A few studies have shown that introducing degradable linkages can impact polymer crystallization kinetics.^41,42,44^ For example, Janani et al. reported the crystallization kinetics of long-spaced aliphatic polyesters and found that polyester with shorter ester spacings (<4 carbons) exhibited lower barriers and a stronger drive to nucleate than those with longer spacings due to stronger ester–ester interactions.^41,45^ However, these systems generally have low molecular weights (∼20 000 g/mol) as well as low melting temperatures (*T*_m_ ∼ 80 °C) and high glass transition temperatures (*T*_g_ ∼ 70 °C) due to the high ester ratios, which make their direct comparison to conventional PE a challenge.

Recently, various research groups have conceptualized the synthesis of PE mimics with in-chain functionalities such as ester and acetal.^44,46–48^ Among these efforts, Li et al. developed a versatile strategy to prepare degradable mimics (DMs) of PE using ring-opening metathesis polymerization (ROMP) and thiol–ene click chemistry.^46^ This approach enabled the synthesis of high molecular weight polymers without restriction on chain length between ester groups. The resulting degradable mimics of high-density polyethylene (HDPE) and linear low-density polyethylene (LLDPE) closely resemble those of their commercial analogues. Importantly, the ester linkages incorporated into their backbones allow polymer degradation into oligomers that can be repolymerized without significantly compromising mechanical and thermal properties. However, the impact of these ester linkages on polymer crystallization kinetics remains unclear, representing a critical knowledge gap for advancing sustainable polymer design and providing information on processing conditions.

This work investigated the crystallization behaviors of degradable HDPE and LLDPE mimics (HDPE-DM and LLDPE-DM) as well as non-degradable HDPE mimics (HDPE-M, absence of both ester groups and short-chain branches), with a focus on understanding their crystallization behaviors. Isothermal and non-isothermal crystallization of PE mimics and commercial PE were analyzed using Avrami, Hoffman–Weeks, Lauritzen–Hoffman, and Kissinger models. We found that the sparse ester incorporation did not affect perfect crystal thermodynamics but reduced the overall crystallization barrier, promoting faster crystallization, likely by serving as the preferred chain-folding sites. Moreover, the crystallization activation energy of HDPE-DM was comparable to that of commercial HDPE. These results can provide key insights for designing sustainable, functional polyolefins.

## Experimental Section

### Materials and Sample Preparation

LLDPE was obtained from ASTM International as part of their proficiency testing program. HDPE was obtained from NOVA Chemicals (SURPASSHPs667). LDPE was obtained from Chevron Phillips Chemical (Marlex1017). All commercial PE samples were purified twice by dissolving them in xylene at 130 °C and then precipitated in methanol to remove possible additives present in the resins. The polymers were collected by gravimetric filtration and dried under vacuum at 40 °C for 24 h. Xylenes (ACS Reagent) and methanol (HPLC grade) were obtained from Fisher Chemical. HDPE-M, HDPE-DM, and LLDPE-DM were prepared according to the previous study.^46^ The values of molecular weight (*M*_n_), polydispersity index (PDI), and ester-to-methylene ratio (E/M) are reported in Table S1. The semicrystalline morphology was characterized by wide-angle X-ray scattering (WAXS) in the previous report.^46^

### General Characterization

For the tensile testing, the samples were melt-pressed into 0.75 mm thick films at 190 °C for a total of 15 min (10 min heating with no pressure and 5 min under 4 MPa) before cooling on an aluminum bench with a steel heat sink on top and holding the mold together for 20 min. Dumbbell-shaped tensile bars were prepared with a die cutter (3.7 mm gauge width and 12.7 mm gauge length). All tensile tests were performed on a Mark-10 F105-EM test frame equipped with a series FS05-50 force sensor with a 250 N capacity at a strain rate of 22 mm min^–1^. The melting and crystallization temperatures (*T*_m_ and *T*_c_) of samples were obtained using differential scanning calorimetry (DSC) via a TA Instruments Discovery DSC250. Tzero pans and lids (from TA Instruments) were used, and a heat–cool–heat cycle was employed with a temperature profile ranging from 25 to 180 °C at a ramp rate of 20 °C min^–1^. Data acquisition and analysis were performed by using TRIOS software by TA Instruments. *T*_c_ and *T*_m_ were determined by the temperature at the peak heat flow from the second and third steps, respectively. The degree of crystallinity (*X*_c_) was determined using the second heating step for all samples, using the following equation:

1 $$X_{c}=\left(\frac{\Delta H_{m}}{293\;J\;g^{-1}}\right)\times 100\%$$

where Δ*H*_m_ represents the enthalpy of fusion (293 J g^–1^ for 100% crystalline PE).

### Isothermal Crystallization Experiments

For isothermal crystallization experiments, the sample was first heated to 180 °C at 20 °C/min to erase the thermal history, then rapidly cooled at 60 °C/min to the crystallization temperature, *T*_c_, followed by holding at *T*_c_ for a crystallization time that was long enough to achieve the saturation of the crystallization, and finally heated at 20 °C/min to 180 °C. These steps were repeated for different *T*_c_ used in this study. The DSC curves recorded during isothermal crystallizations at different temperatures and the subsequent DSC heating scans of the melt-crystallized samples are reported in Figures S1–S6.

The data obtained from the experiments of isothermal crystallization were analyzed with the Avrami theory, following the established guidelines.^49,50^ The Avrami equation can be expressed as^51,52^

2 $$X\left(t\right)=1-\exp\left(-Kt^{n}\right)$$

or

3 log[−ln(1−X(t))]=log⁡K+n⁡log⁡t

where *K* is the Avrami constant and describes the overall crystallization rate constant, *n* is the Avrami exponent and is related to the time dependence of nucleation (*n*_n_) and crystal growth geometry (*n*_gD_), as expressed:

4 $$n=n_{\mathrm{gD}}+n_{n}$$

The *n*_gD_ values can only be integer numbers, 1, 2, and 3, corresponding to one-, two-, and three-dimensional entities, respectively. The *n*_n_ contribution can take a value from 0 to 1, where 0 corresponds to instantaneous nucleation and 1 corresponds to sporadic nucleation.^49,53,54^ *X*(*t*) is the instantaneous relative crystallinity at time *t*, calculated as

5 $$X\left(t\right)=\frac{Q_{t}}{Q_{\infty }}=\frac{\int_{0}^{t}\left(\frac{dH}{dt}\right)dt}{\int_{0}^{\infty }\left(\frac{dH}{dt}\right)dt}$$

Here, *Q*_*t*_ is the instantaneous heat flow, *Q*_∞_ is the total heat flow throughout the crystallization process, and d*H*/d*t* is the instantaneous enthalpy change rate. *X*(*t*) as a function of *t* is shown in Figure S7. To ensure that there is no impingement between crystals and the approximation of the Avrami theory is valid, the isothermal crystallization data were fitted in the conversion range between 3 and 20%.^50^ The values of *K* and *n* can be determined from the intercept and slope, respectively, of the Avrami plot of log­[−ln­(1 – *X*(*t*))] vs log *t*. *K* and *n* of isothermal crystallization are reported in Figure S8.

The equilibrium melting temperatures $\left(T_{m}^{0}\right)$ of all samples were evaluated with the Hoffman–Weeks extrapolation in the plots of the melting temperature as a function of the isothermal crystallization temperature (Figure S9).^55^ These data were fitted to a straight line in a graph of *T*_m_ vs *T*_c_, where the line was extrapolated to its intersection with the line *T*_m_ = *T*_c_. The temperature at this intersection was $T_{m}^{0}$ (Table S1).

The values of half-crystallization time *t*_1/2_, which represent the time needed for the crystallization of 50% of the crystallizable material, were obtained from the kinetic curves of *X*(*t*) vs *t*. The reciprocal of the half-crystallization time *t*_1/2_ was taken as an approximation of the overall crystallization rate that includes both nucleation and growth contributions to be fitted with the Lauritzen–Hoffman (L–H) equation^56,57^ using the Origin App developed by Müller et al.^49^

The L–H theory describes the growth of polymer lamellae and secondary nucleation, which provides analytical expressions for the linear spherulitic growth rate as a function of supercooling $\left(\Delta T=T_{m}^{0}-T_{c}\right)$ . This model has been adapted to fit overall crystallization rates obtained by DSC (i.e., including primary and secondary nucleation).^5^ The data were fitted assuming crystallization in Regime II.^58^ The generalized L–H expression is presented as

6 $$\frac{1}{t_{1/2}}=G_{0}\exp\left(-\frac{U^{*}}{R\left(T_{c}-T_{\infty }\right)}\right)\exp\left(-\frac{K_{g}}{T_{c}\Delta Tf}\right)$$

or

7 $$\ln\frac{1}{t_{1/2}}=\ln G_{0}-\frac{U^{*}}{R\left(T_{c}-T_{\infty }\right)}-\frac{K_{g}}{T_{c}\Delta Tf}$$

where *G*_0_ is a prefactor that incorporates the growth rate dependence on structural factors other than temperature, U* is the activation energy for the transport of a polymer chain from the melt to the crystal surface, *R* is the gas constant (8.314 J mol^–1^ K^–1^), *T*_∞_ is a hypothetical temperature at which chain mobility ceases, and it is usually taken as *T*_∞_ = *T*_g_ – 30­(K), *f* $\left(f=2T_{c}/(T_{c}+T_{m}^{0})\right)$ is a temperature correction term accounting for the change in the enthalpy of melting with crystallization temperature, and *K*_g_ is the thermodynamic nucleation barrier that can be expressed as

8 $$K_{g}=\frac{2b_{0}\sigma \sigma_{e}T_{m}^{0}}{k\Delta H_{m}^{0}}$$

where $\Delta H_{m}^{0}$ is the heat fusion of a perfect crystal, *k* is the Boltzmann constant (1.38 × 10^–23^ J K^–1^), *b*_0_ is the thickness of a polymer stem, σ is the lateral surface free energy, and σ_e_ is the fold surface free energy. The parameters used for the L–H fitting were taken from the literature.^5,59^ The L–H fitting and all parameters are shown in Figure S10.

### Non-isothermal Crystallization Kinetics

For non-isothermal crystallization kinetics, the samples were first heated to 180 °C at 20 °C/min and held isothermally for 1 min to erase the thermal history. Then, the sample was cooled to 40 °C at various cooling rates (Φ). The cycle was repeated five times with Φ = 2, 5, 10, 20, and 40 °C/min.

The Avrami model can be extended to analyze non-isothermal crystallization using eq 9

9 $$X\left(t\right)=1-\exp\left(-K^{\prime }t^{n^{\prime }}\right)$$

where *K*′ and *n*′ are the Avrami constant and the Avrami exponent for non-isothermal crystallization, respectively, and the crystallization time (*t*) is,

10 $$t=\frac{T_{\mathrm{onset}}-T}{\phi }$$

where *T*_onset_ is the onset temperature of the crystallization process and ϕ is the cooling rate. Due to the crystallization kinetic measurements being non-isothermal, it has been established that *K* should be corrected to obtain the Jeziorny-modified Avrami constant (*K*′) at a normalized cooling rate using eq 11

11 $$\log K^{\prime }=\frac{\log K}{\phi }$$

The crystallization activation energy was determined using the Kissinger equation:^60^

12 $$\ln\left(\frac{\phi }{{T_{P}}^{2}}\right)=C-\frac{\Delta E}{RT_{P}}$$

where *C* is a constant, *R* is the gas constant, *T*_p_ is the peak crystallization temperature in K, and Δ*E* is the activation energy.

## Results and Discussion

### Thermal and Mechanical Properties of HDPE, LLDPE, and Their Degradable Mimics

HDPE and LLDPE are two widely used polyolefins. Their molecular structures (i.e., linear vs branched) are illustrated in Figure 1A, where HDPE is shown as a linear polymer with very few short-chain branches, while LLDPE contains regular short-chain branches introduced through copolymerization with long-chain α-olefins. The chemical structures of HDPE-DM, LLDPE-DM, and the degradation/repolymerization pathways are presented in Figure 1B. Leveraging the ROMP of cis-cyclooctene and lactone, linear backbones can be synthesized without branching. Furthermore, short-chain branching can be introduced through thiol–ene chemistry, yielding a polyolefin architecture closely resembling LLDPE. The DSC curves recorded from the second heating at 20 °C/min of HDPE, LLDPE, and their degradable mimics are reported in Figure 2A, with the corresponding melting temperatures (*T*_m_) and degree of crystallinity (*X*_c_) summarized in Table S1. LLDPE-DM (*T*_m_ = 120.8 °C, *X*_c_ = 47%) exhibited a melting temperature nearly the same as the commercial LLDPE (*T*_m_ = 121.5 °C), with a slightly higher crystallinity (*X*_c_ of commercial LLDPE in this study is approximately 34%). HDPE-DM (*T*_m_ = 128.7 °C, *X*_c_ = 51.2%) had both a lower melting temperature and crystallinity than that of commercial HDPE used in this study (*T*_m_ = 134 °C, *X*_c_ = 80.9%). While the crystallinity of HDPE-DM and LLDPE-DM differed from those of the commercial PE examined here, the values remained within the reported ranges for HDPE (*X*_c_ = 60–80%) and LLDPE (*X*_c_ = 30–50%).^6^ HDPE-DM exhibited slightly lower crystallinity than the non-degradable analog HDPE-M (*X*_c_ = 60%, Table S1), which can be attributed to the local chain irregularities introduced by the ester linkages. The corresponding mechanical properties were investigated by uniaxial tensile testing and summarized in Figure 2B.^46^ HDPE-DM exhibits an average Young’s modulus of 686 MPa, a yield stress of 22.9 MPa, and a strain at break of 458%, while those values for commercial HDPE are 674 MPa, 19.4 MPa, and 204%, respectively. We attribute the difference in strain at break between the samples to variations in crystalline lamellar thickness, as evidenced by their distinct melting temperatures (*T*_m_). Additionally, average Young’s modulus and yield stress of LLDPE-DM (205 and 11.2 MPa) were comparable to those of commercial LLDPE (150 and 9.9 MPa).

**Figure 1:**
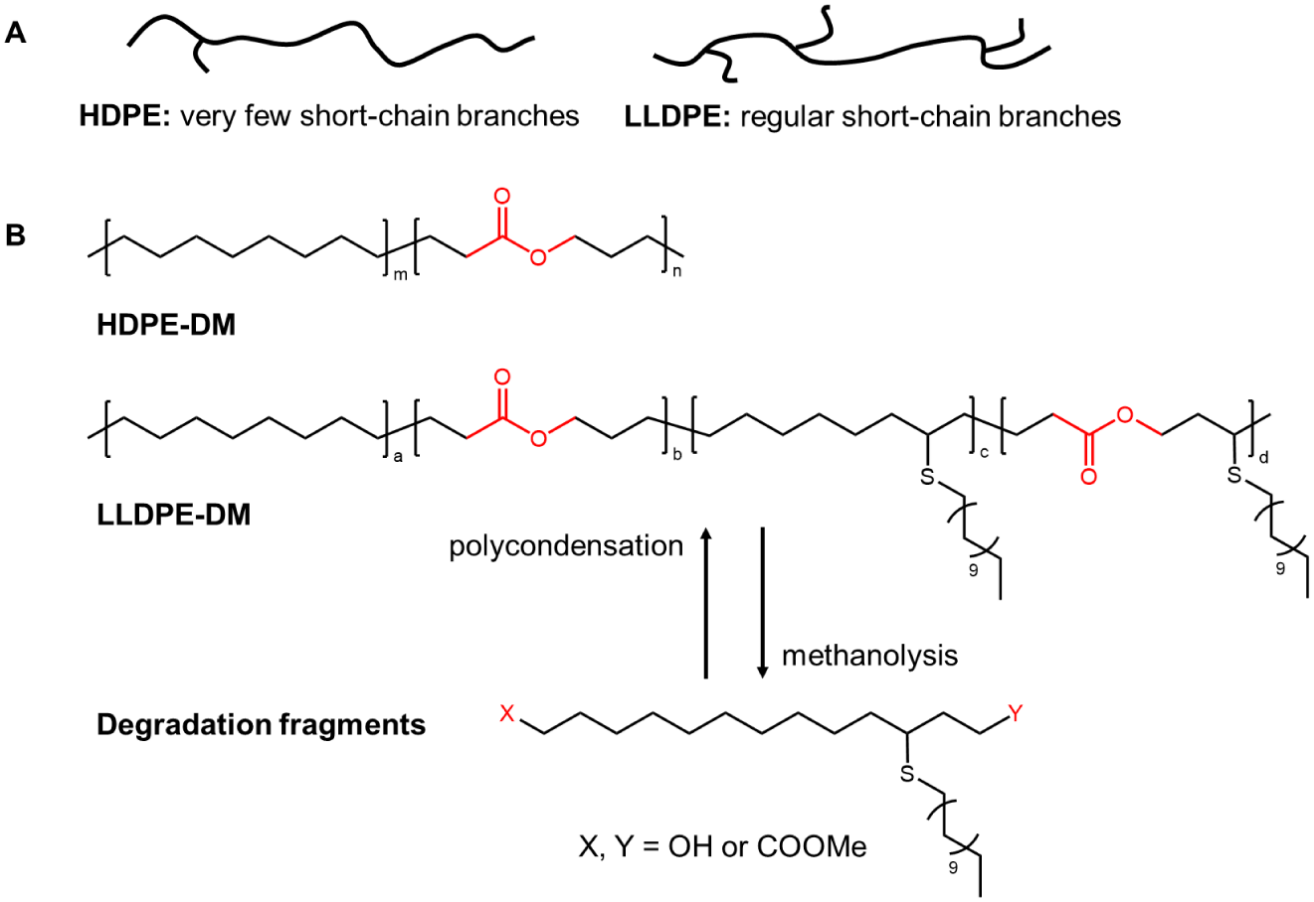
(A) Schematic illustration of HDPE and LLDPE, and (B) chemical structures of HDPE-DM and LLDPE-DM, and the recycling route of LLDPE-DM.

**Figure 2:**
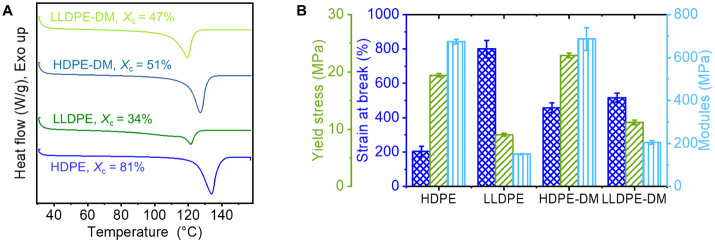
(A) DSC curves of the second heating recorded at 20 °C/min. (B) Young’s modulus, yield stress, and strain at break of HDPE, LLDPE, HDPE-DM, and LLDPE-DM.

### Isothermal Crystallization Kinetics

Commercial HDPE, commercial LLDPE, HDPE-DM, and LLDPE-DM samples were isothermally crystallized from the melt at different crystallization temperatures (*T*_c_) to analyze the effect of degradable ester linkages on the crystallization kinetics of HDPE and LLDPE. The isothermal crystallization experiments were carried out in DSC, and the obtained data were analyzed with Avrami’s model^51,52^ and Lauritzen–Hoffman (L–H) theory.^56,57^ Figure 3A–D shows selected exotherms capturing complete isothermal crystallization. In contrast, LLDPE exhibited a narrower range of crystallization temperatures and partially incomplete exotherms at higher *T*_c_ (120–121 °C), suggesting that a small fraction of crystallization occurred during the cooling step. As expected, higher *T*_c_ led to longer crystallization time, which is a typical behavior at low supercooling (Δ*T*) for semicrystalline polymers.^50^ A general trend was found in which commercial HDPE and LLDPE crystallized faster than their corresponding mimics, as reflected by their higher *T*_c_ values. HDPE-M (hydrogenated ROMP poly­(cyclooctene)) (Figure S11B) and HDPE-DM exhibited comparable *T*_c_, suggesting that the small fraction of ester groups in the DM samples did not significantly contribute to the reduced crystallization temperature of the mimics, consistent with previous computational studies.^46^ Because HDPE-M shows a depressed equilibrium melting point $\left(T_{m}^{0}\right)$ yet similar supercooling at equal crystallization half-time relative to HDPE (Table S1, which will also be discussed in the following section), the lower isothermal crystallization temperature of HDPE-M is potentially attributed to thermodynamic effects from the presence of subtle microstructural defects in these polyethylene mimics.

**Figure 3:**
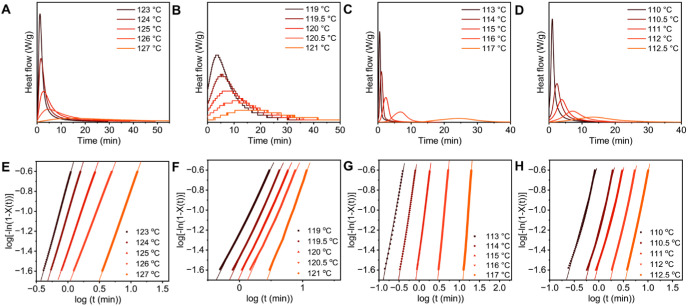
Representative isothermal crystallization exotherms at different crystallization temperatures for (A) HDPE, (B) LLDPE, (C) HDPE-DM, and (D) LLDPE-DM, and linear fit of the Avrami plot in the range of 3% ≤ *X*(*t*) ≤ 20% for (E) HDPE, (F) LLDPE, (G) HDPE-DM, and (H) LLDPE-DM.

The development of relative crystallinity as a function of time during isothermal crystallization of the PE samples was determined using eq 3 and is shown in Figure S7. The corresponding Avrami plots are shown in Figure 3E–H, where the solid lines are linear fits of eq 5 to the experimental data. To comply with the free growth approximation, crystallinity (*X*(*t*)) between 3% and 20% was used for fitting.^49^ In all cases, the isothermal crystallization data can be described by the Avrami equation. The resulting Avrami exponents (*n*), obtained from the slope of the Avrami plots, were summarized as a function of crystallization temperature in Figure 4A. Commercial HDPE exhibited *n* ≈ 2 across the isothermal crystallization window, consistent with instantaneous nucleation (all nuclei formed at the onset of crystallization) and predominantly two-dimensional spherulitic growth. In contrast, HDPE-DM exhibited higher and temperature-dependent Avrami exponents that ranged from ∼2 to >4 with increasing *T*_c_, indicating that nucleation became more sporadic and the crystal growth approached three-dimensional.^61,62^ This result suggested that nucleation cannot be fully activated at the onset under low supercooling, which might be attributed to the broad distribution of crystallizable sequence lengths created by ester linkages. At lower *T*_c_ (higher supercooling), most of the sequences can nucleate immediately, resulting in crystalline regions that incorporate both alkyl segments and ester groups, in agreement with previous computational results.^46^ However, at higher *T*_c_ (lower supercooling), only the most regular and longest sequences can nucleate at the onset, while the shorter sequences become active later in time.^61^ At the highest *T*_c_ (117 °C, Δ*T* = 14 °C) examined in this work, *n* of HDPE-DM exceeded 4, consistent with the models describing time-dependent nucleation rates.^63^ Although LLDPE-DM maintained *n* ≈ 2 across its crystallization window, a slight increase was observed at higher *T*_c_, suggesting a gradual shift from instantaneous to more sporadic nucleation, similar to the trend observed for HDPE-DM. Since no crystallization exotherms were detected after 1 h at higher temperatures (e.g., 112.5 °C, Figure S4), the isothermal crystallization under very low supercooling (Δ*T* < 17 °C) was not examined. This limitation implies that LLDPE-DM may nucleate even more slowly than HDPE-DM under equivalent supercooling conditions. LLDPE exhibited a low *n* ≈ 1–2, indicative of predominantly one-dimensional growth. Given the time-dependent nucleation behavior observed in all PE mimics (Figure S8A), the slightly higher *n* of LLDPE-DM (2–2.5) likely reflected a one-dimensional growth combined with sporadic nucleation. The Avrami constant *K* was obtained from the intercept of the Avrami plots in Figure 3. For comparison across samples, *K* ^1/*n*^ was plotted as a function of crystallization temperature in Figure 4B.^5,49^ The half-time of crystallization, *t*_1/2_, which represents the time to reach 50% crystallinity, was extracted from the kinetic curves in Figure S7. The inverse of half-crystallization time 1/*t*_1/2_ gives the experimental overall crystallization rate, which includes contributions of both nucleation and growth. The trends of the Avrami constant *K* ^1/*n*^ were very similar to those of 1/*t*_1/2_ reported in Figure S13, confirming that the kinetic parameters derived from the isothermal crystallization curves primarily reflect crystal growth during the isothermal stage. For all DMs and commercial PE samples, *K* ^1/*n*^ decreased with increasing crystallization temperature, which implied a slower overall crystallization rate at higher *T*_c_.

**Figure 4:**
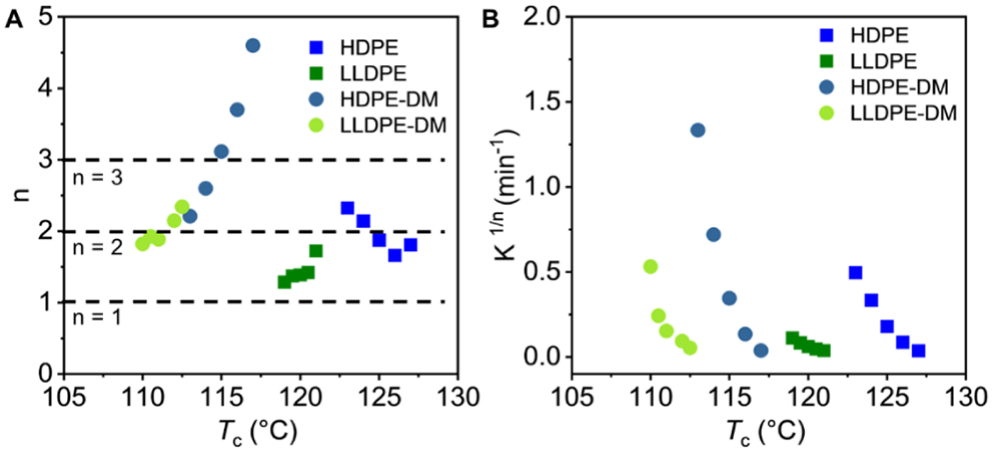
(A) Avrami exponent *n* and (B) Avrami constant *K* for HDPE, LLDPE, HDPE-DM, and LLDPE-DM.

To assess how supercooling affects crystallization rates, we calculated the equilibrium melting temperatures $\left(T_{m}^{0}\right)$ of PE samples using the Hoffman–Weeks extrapolation method.^55^ Plots of melting temperatures versus crystallization temperatures for each PE (Figures S1–S6) are shown in Figure 5A. $T_{m}^{0}$ was obtained by linear extrapolation of the *T*_m_ – *T*_c_ linear fits to their intersection with *T*_m_ = *T*_c_, and the resulting values are summarized in Table S1. The equilibrium melting temperatures of commercial HDPE and LLDPE are 141.5 and 135.7 °C, respectively, consistent with the literature values.^64,65^ HDPE-DM $\left(T_{m}^{0}=131.5^{{}^{\circ}}C\right)$ and LLDPE-DM $\left(T_{m}^{0}=129.5^{{}^{\circ}}C\right)$ exhibited lower $T_{m}^{0}$ than their commercial PE counterparts. By contrast, HDPE-M showed a $T_{m}^{0}$ (131.1 °C, Figure S9 and Table S1) comparable to HDPE-DM, suggesting that the small fraction of ester groups in the DM samples did not measurably change the perfect crystal thermodynamics.^66^ Moreover, HDPE-M also showed a depressed $T_{m}^{0}$ relative to HDPE, suggesting that the lower $T_{m}^{0}$ of PE mimics relative to their commercial counterparts likely reflected subtle defects intrinsic to the PE mimics, such as possible incomplete hydrogenation^46^ or occasional formation of cyclic segments from backbiting side reactions.^67^

**Figure 5:**
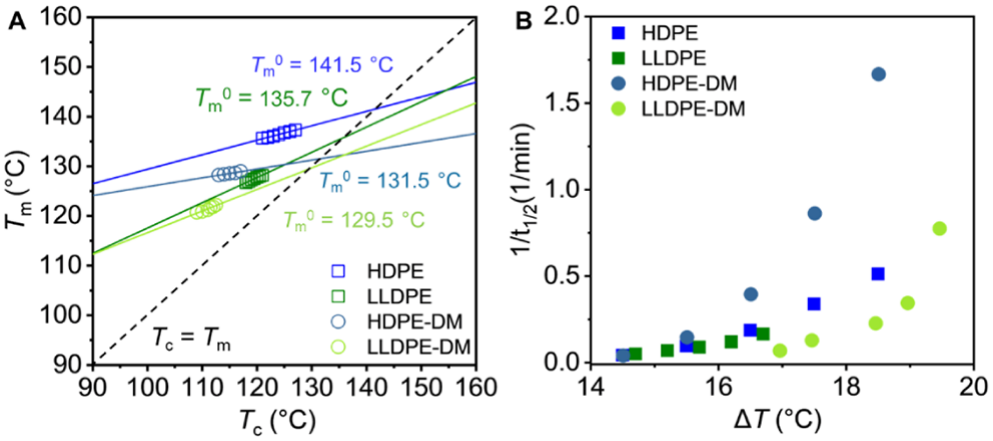
(A) Hoffmann–Weeks linear fit of melting temperatures (*T*_m_) as a function of the crystallization temperature (*T*_c_) and (B) overall crystallization rate as the inverse of the half crystallization time (1/*t*_1/2_) as a function of the supercooling $\left(\Delta T=T_{m}^{0}-T_{c}\right)$ for HDPE, LLDPE, HDPE-DM, and LLDPE-DM.

The overall isothermal crystallization rate, expressed as the inverse of the half-crystallization time (1/*t*_1/2_) as a function of the supercooling $\left(\Delta T=T_{m}^{0}-T_{c}\right)$ , is plotted in Figure 5B for PE samples. As anticipated, LLDPE required slightly higher supercooling than HDPE to achieve comparable crystallization rates, which can be attributed to the kinetic limitations from short-chain branching hindrance, requiring a greater thermodynamic driving force to overcome them.^68–70^ A similar but more pronounced trend was observed between HDPE-DM and LLDPE-DM as well. Interestingly, for higher supercooling (Δ*T* > 16 °C), HDPE-DM crystallized faster than commercial HDPE or HDPE-M (Figure S9) at the same Δ*T*, whereas their crystallization rates were similar when Δ*T* < 16 °C. Moreover, the Lauritzen–Hoffman (L–H) analysis showed that HDPE-DM had a larger growth prefactor *G*_0_ (the growth rate dependence on structural features other than temperature) than HDPE (HDPE-DM: 1.47 × 10^6^, HDPE: 3.57 × 10^3^; Figure S10), suggesting that HDPE-DM has a higher interfacial attachment frequency.^56,57^ This enhancement might be attributed to the ester group located near the crystalline surface, which promoted segregation of polar moieties to the fold surface, thereby improving the probability of successful chain registration.^41,45,48,71^ This explanation can be further supported by previous computational results, indicating that an average of 45 mol % ester groups were present in the crystalline region for HDPE-DM.^46^

### Non-isothermal Crystallization Kinetics

Since most industrial processing conditions for PE are non-isothermal, it is also crucial to study the non-isothermal crystallization kinetics of the degradable mimics of PE.^72^ The non-isothermal crystallization thermograms of commercial HDPE, LLDPE, and their degradable mimics are shown in Figure 6A–D, with cooling rates (Φ) of 2, 5, 10, 20, and 40 °C/min. As expected, as the cooling rate increased, both the onset (*T*_onset_) and peak (*T*_p_) crystallization temperatures decreased (Table S2). Meanwhile, the exothermic peaks became broader with increasing cooling rates due to the formation of imperfect crystal structures upon rapid cooling.^14,17,73^ The supercooling needed for each sample at each cooling rate was obtained by $\Delta T=T_{m}^{0}-T_{p}$ and is summarized in Table S2. At the same cooling rate in the range from 2 °C min^–1^ to 40 °C min^–1^, the degradable mimics of PE consistently exhibited lower Δ*T* values than their corresponding commercial PE samples, indicating that the mimics required less undercooling to reach the maximum crystallization rate during a continuous cooling process. This trend was consistent with the isothermal results, where HDPE-DM crystallized faster than commercial HDPE under higher supercooling temperatures, suggesting that the ester linkages facilitate overall crystallization kinetics.

**Figure 6:**
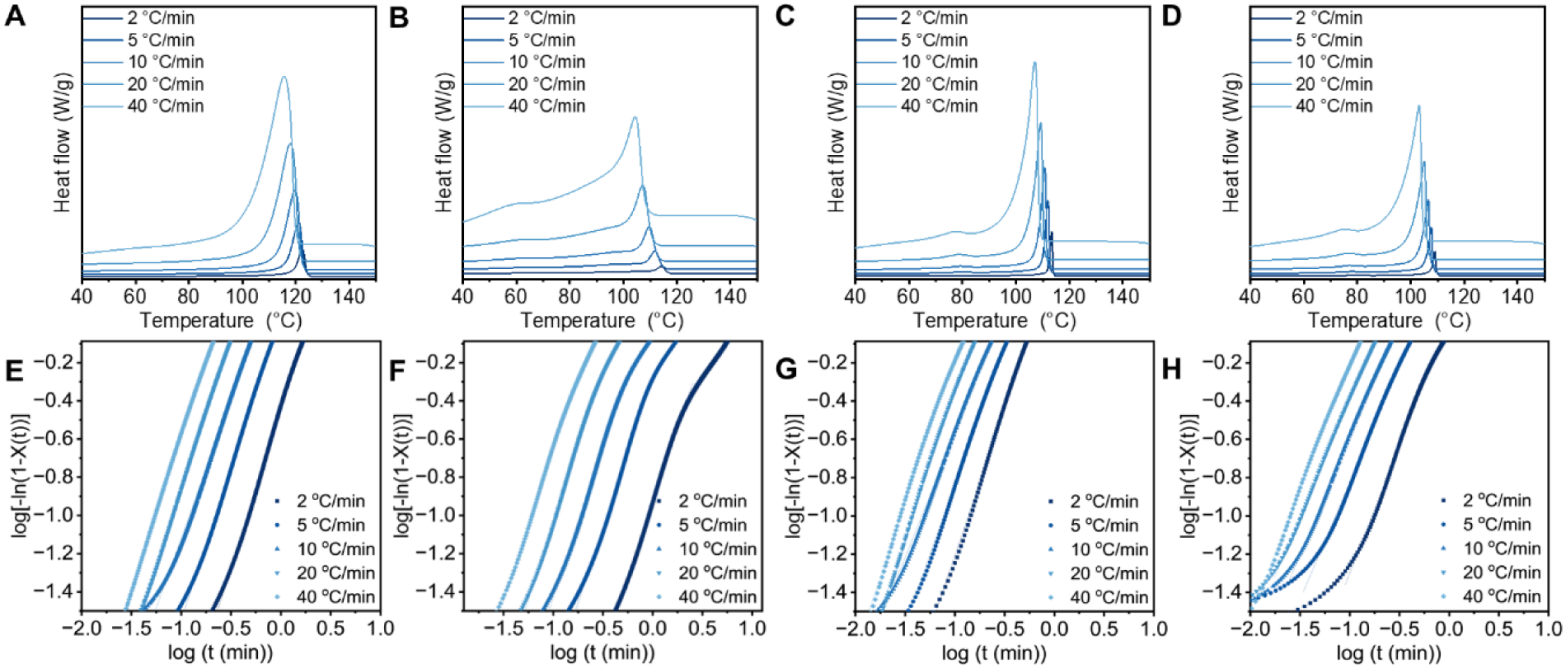
Representative non-isothermal crystallization exotherms at different cooling rates for (A) HDPE, (B) LLDPE, (C) HDPE-DM, and (D)­LLDPE-DM, and the Avrami plot in the range of 20% ≤ *X*(*t*) ≤ 60% for (E) HDPE, (F) LLDPE, (G) HDPE-DM, and (H)­LLDPE-DM.

The non-isothermal crystallization kinetics were further analyzed by the Avrami model.^5,14,17,72,74–80^ The rearranged form of the Avrami equation used to generate the Avrami plots for non-isothermal crystallization can be found in the Experimental Section. Equation 5 was utilized to calculate the instantaneous relative crystallinity (*X*(*t*), Figure S14) and eq 10 was used to convert the crystallization temperatures to the crystallization time (*t*). For non-isothermal crystallization, Avrami analysis was performed in the intermediate relative crystallinity range (20% ≤ *X*(*t*) ≤ 60%), where the model assumptions are most valid without the influence of early nucleation or secondary crystallization.^50,80,81^ As in isothermal crystallization, both the Avrami exponent and the Avrami constant can be extracted from the slope and intercept of the linear region, where the relevant equation applies. To reflect the intrinsic crystallization kinetics, it has been established that the rate constant *K* should be corrected to the Jeziorny-modified Avrami constant (*K*′) using eq 11. The resulting Avrami exponents (*n*′) and Jeziorny-modified Avrami constants (*K′*) for PE samples are summarized as a function of cooling rate (ϕ) in Figure 7. As shown in Figure 7A, for all PE samples, *n*′ ranged between 1 and 2 and remained nearly constant across cooling rates, implying one-dimensional growth and heterogeneous nucleation. Because non-isothermal conditions were used, the *n*′ and *K*′ values were different from those obtained under isothermal conditions (Figure 4A).^72,82,83^ It was observed that at low cooling rates (ϕ ≤ 10 °C/min), the DM samples exhibited higher *K*′ than their corresponding commercial PE samples, indicating that the incorporation of ester linkages facilitated faster overall crystallization when sufficient time was provided to allow chain re-organization during cooling, consistent with the observation from isothermal crystallization. As the cooling rate increased (ϕ >10 °C/min), the *K*′ values of all samples began to converge. This convergence was attributed to increased kinetic constraints at rapid cooling, where higher supercooling reduced chain mobility and limited the time available for chain packing, thereby diminishing the structural influence.

**Figure 7:**
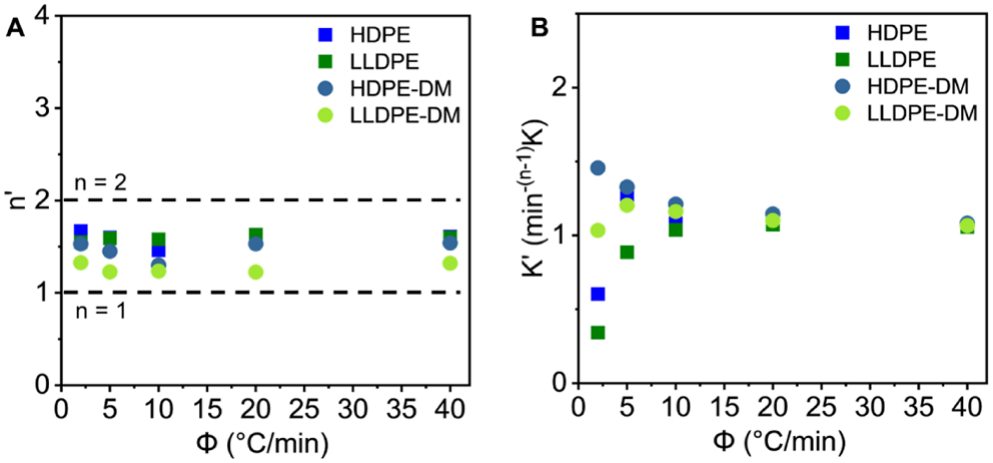
(A) Avrami exponent *n*′ and (B) Jeziorny-modified Avrami constant *K*′ as a function of cooling rate (ϕ) for HDPE, LLDPE, HDPE-DM, and LLDPE-DM.

The Kissinger method was used to determine the crystallization activation energies (Δ*E*) of all PE samples.^60^ In non-isothermal analysis using DSC measurement results, the apparent Δ*E* includes contributions from both the nucleation activation energy (free energy of formation of crystal nuclei with a critical size) and transport activation energy (transport of polymer segments across the phase boundary to the crystal growth surface).^84^ The resulting plot is shown in Figure 8, where Δ*E* was determined from the slope and is labeled in the figure. Literature reports a broad range (300–540 kJ/mol) of HDPE crystallization activation energies, and this can be due to several factors, including polymer molecular weight and the presence of additives.^85–89^ The Δ*E* of commercial HDPE in this work (556 kJ/mol) was on the upper end of the literature-reported range, likely because the pretreatment (see the Experimental Section) of the commercial samples removed some additives that can impact crystallization kinetics. Consistent with this interpretation, the unprecipitated commercial HDPE shows a much lower Δ*E* (285 kJ/mol, Figure S17). The precipitation process did not significantly change Δ*E* for commercial LLDPE, which might be because the original sample does not contain additives (i.e., nucleating agents). HDPE-DM (Δ*E* = 584 kJ/mol) exhibited a comparable crystallization activation energy to the commercial HDPE. Notably, the ester-free analog HDPE-M showed a higher Δ*E* (671 kJ/mol; Table S1 and Figure S18) than HDPE-DM, indicating that incorporating ester linkages reduced the apparent overall crystallization barrier. This trend was consistent with the observed faster crystallization kinetics and larger growth prefactor *G*_0_ for HDPE-DM relative to HDPE. LLDPE-DM exhibited a slightly higher Δ*E* (606 kJ/mol) than HDPE-DM, consistent with added short-chain branching imposing steric constraints that raise the apparent barrier.^90,91^

**Figure 8:**
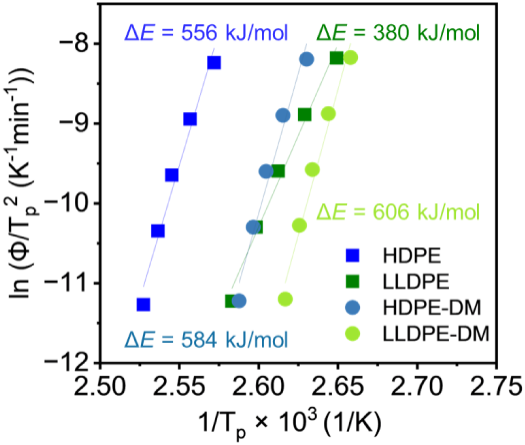
Kissinger plot for HDPE, LLDPE, HDPE-M, HDPE-DM, and LLDPE-DM.

## Conclusions

In this work, we systematically studied the crystallization behaviors of degradable HDPE and LLDPE mimics and compared their isothermal and non-isothermal crystallization behaviors with commercial PE using Avrami analysis, Hoffman–Weeks extrapolation, Lauritzen–Hoffman (L–H) theory, and Kissinger models. Equilibrium melting temperatures obtained by Hoffman–Weeks extrapolation showed that the ester functionality did not alter the perfect crystal thermodynamics of the degradable PE mimics. For non-isothermal crystallization, all samples displayed primarily one-dimensional growth with heterogeneous nucleation, with limited influence from ester linkages or short-chain branching on growth dimensionality. For isothermal crystallization, the PE mimics showed more time-dependent nucleation than commercial PE samples, potentially due to a reduced availability of effective nuclei in PE mimics. HDPE-DM exhibited higher overall crystallization kinetics than non-degradable HDPE at the same supercooling once Δ*T* was sufficiently large (>16 °C). L–H analysis showed a much larger growth prefactor *G*_0_ for HDPE-DM than for non-degradable HDPE (HDPE-M), consistent with enhanced interfacial attachment, likely arising from a higher density of effective attachment sites associated with ester linkages at or near the fold surface. The crystallization activation energy of HDPE-DM was comparable to commercial HDPE and lower than that of ester-free HDPE-M, suggesting that the ester linkage reduced the apparent overall crystallization barrier. While this study focuses on model degradable PEs with spaced ester linkers, the distribution of these linkages may also influence crystallization behavior, because statistically incorporated esters can disrupt chain regularity, which may require further investigation in future work. These findings elucidated how sparse polar ester linkages influence the crystallization behaviors of degradable PE mimics and provided fundamental insight for materials and process design for sustainable alternatives to functional polyolefin materials.

## Supplementary Material



## Notes

DSC curves of crystallization exotherms, relative crystallinity as a function of time, and Avrami parameters for isothermal and non-isothermal crystallization, Hoffmann–Weeks linear fit of melting temperatures as a function of the crystallization temperature, inverse of the half-crystallization time from isothermal crystallization, peak crystallization temperature during non-isothermal crystallization, and the Kissinger plot of corresponding samples (PDF)
