# Supplementary material for: Impact of Degradable Linkages on the Crystallization Behaviors of Polyethylene Mimics
Source: Macromolecules. 2025 Nov 21;58(23):12716–26. doi: 10.1021/acs.macromol.5c02706 (PMC12874649; doi:10.1021/acs.macromol.5c02706)
Supplement: Supplementary file 1 [file ma5c02706_si_001.pdf]

Supporting Information for:

Impact of Degradable Linkages on the Crystallization Behaviors of Polyethylene Mimics

Jin Qian [a], Xiaomeng Li [b], Chuanbing Tang [a], Zhe Qiang[,b]*

[a] School of Polymer Science and Engineering, The University of Southern Mississippi, 118 College Drive, Hattiesburg, MS 39406

[b] Department of Chemistry and Biochemistry, University of South Carolina, Columbia, South Carolina 29208, United States

*Corresponding Author:

Zhe Qiang (Email: zhe.qiang@usm.edu)

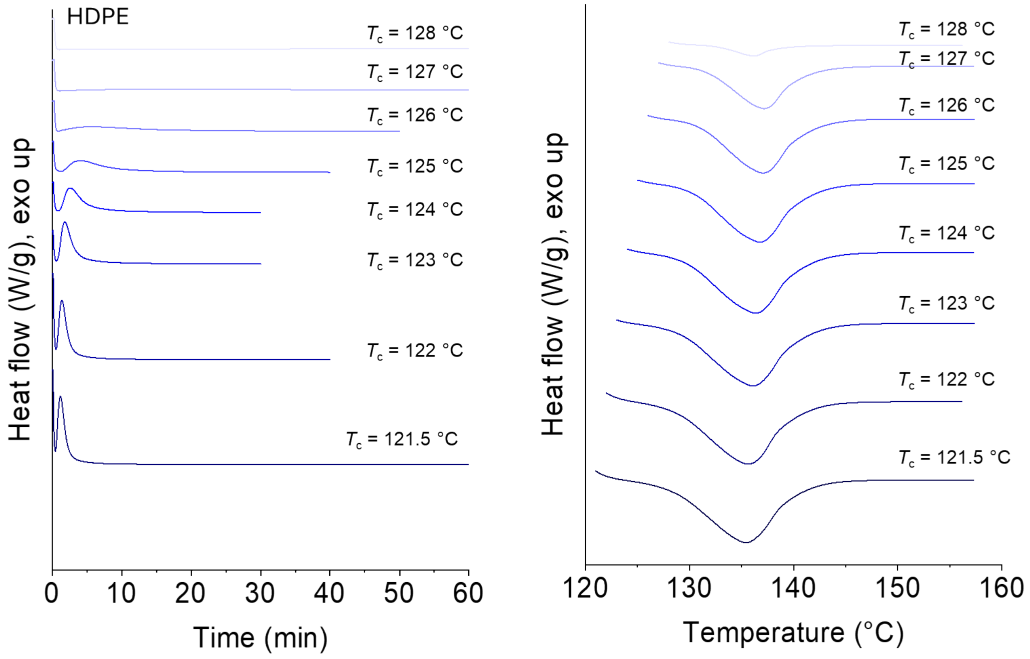

Figure S1. DSC curves of crystallization exotherm as a function of the crystallization time at different isothermal crystallization temperatures (left), and heating curves recorded at 20 °C/min after isothermal crystallization of HDPE (right).

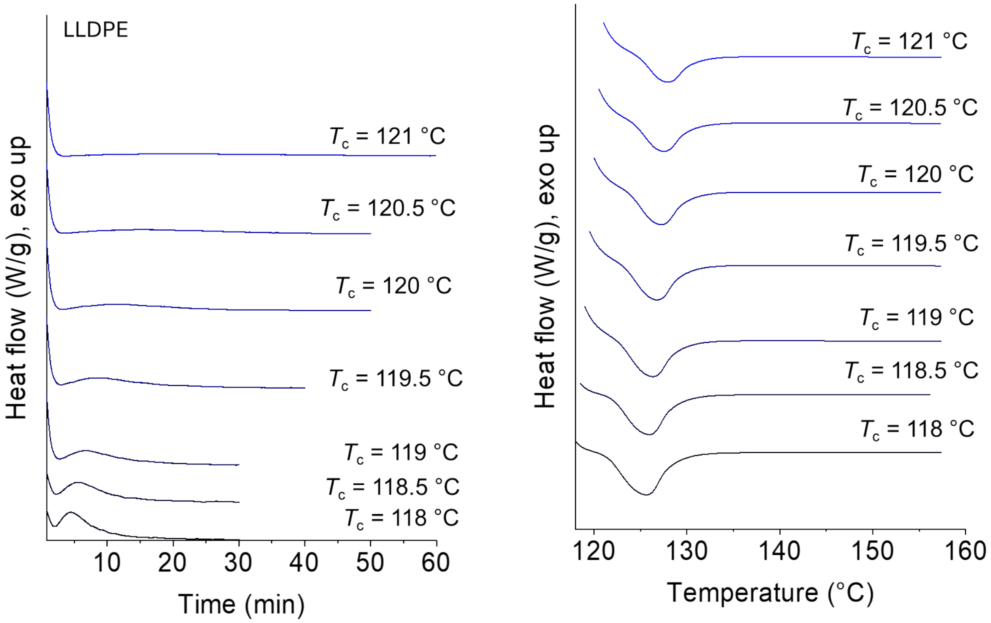

Figure S2. DSC curves of crystallization exotherm as a function of the crystallization time at different isothermal crystallization temperatures (left), and heating curves recorded at 20 °C/min after isothermal crystallization of LLDPE (right).

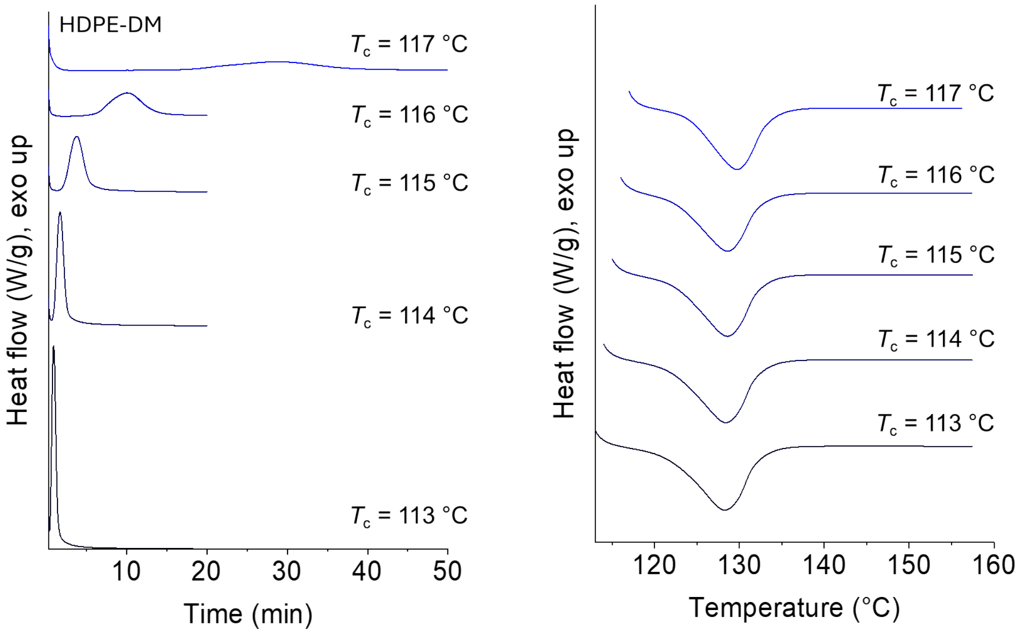

Figure S3. DSC curves of crystallization exotherm as a function of the crystallization time at different isothermal crystallization temperatures (left), and heating curves recorded at 20 °C/min after isothermal crystallization of HDPE-DM (right).

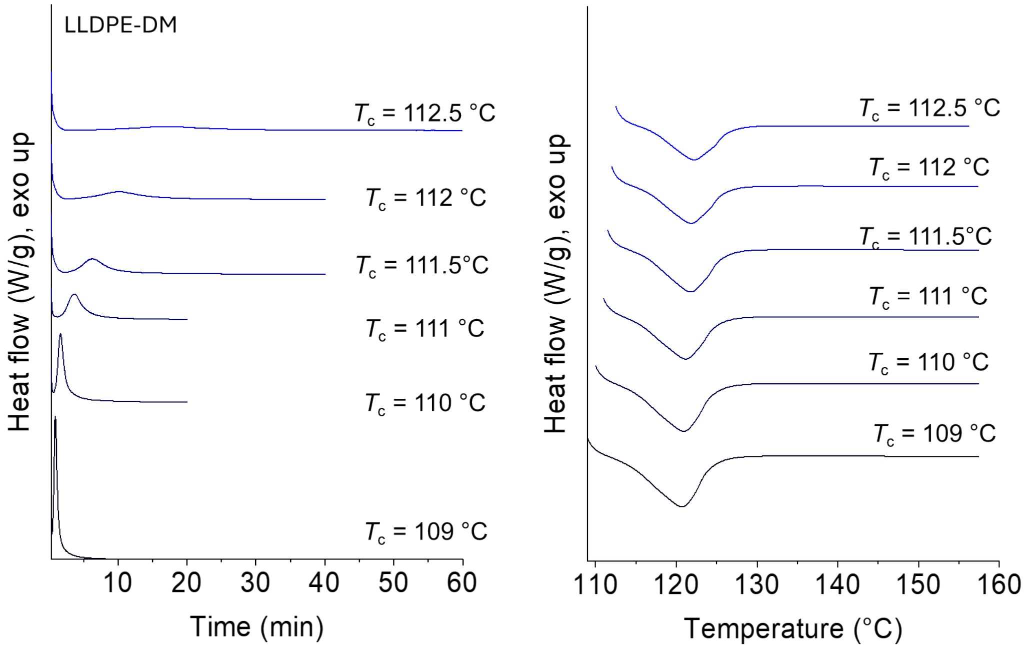

Figure S4. DSC curves of crystallization exotherm as a function of the crystallization time at different isothermal crystallization temperatures (left), and heating curves recorded at 20 °C/min after isothermal crystallization of LLDPE-DM (right).

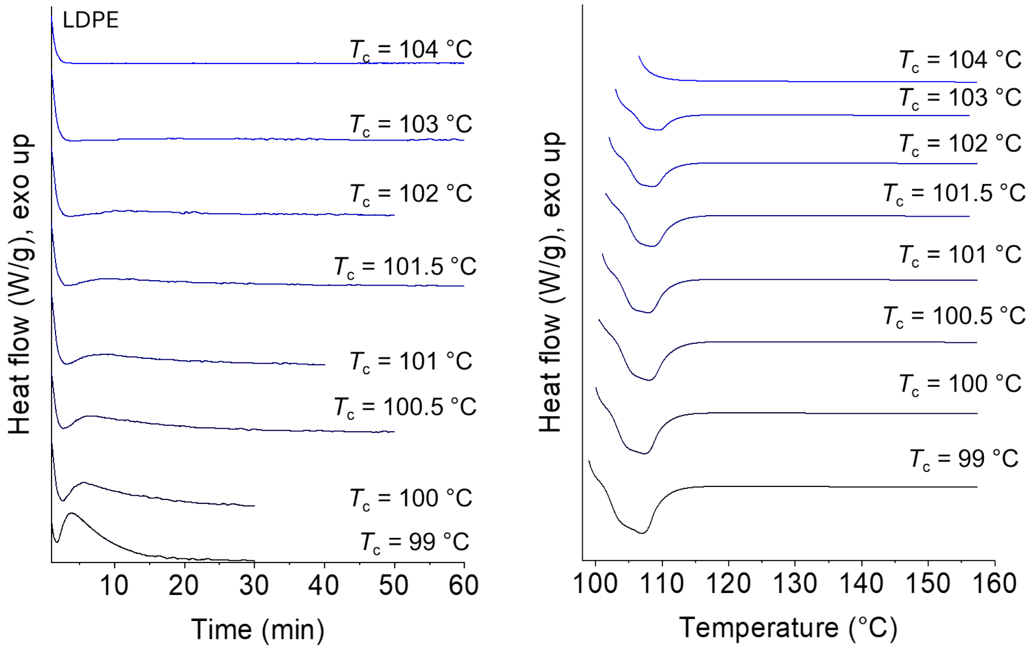

Figure S5. DSC curves of crystallization exotherm as a function of the crystallization time at different isothermal crystallization temperatures (left), and heating curves recorded at 20 °C/min after isothermal crystallization of LDPE (right).

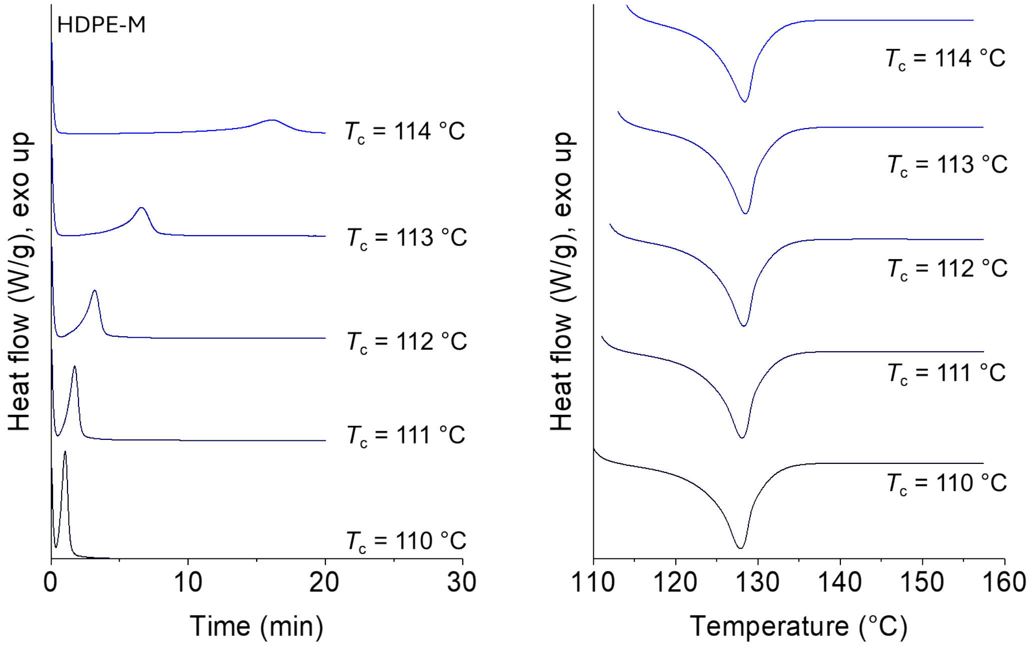

Figure S6. DSC curves of crystallization exotherm as a function of the crystallization time at different isothermal crystallization temperatures (left), and heating curves recorded at 20 °C/min after isothermal crystallization of HDPE-M (right).

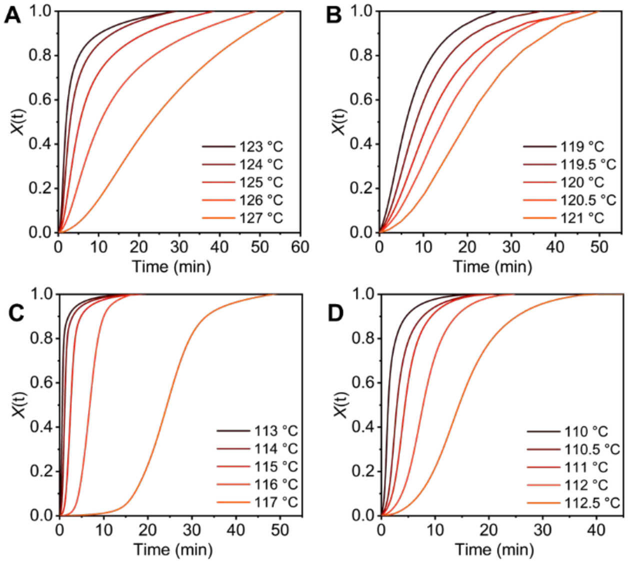

Figure S7. Relative crystallinity as a function of time for the isothermal crystallization of (A) HDPE, (B) LLDPE, (C) HDPE-DM and (D)LLDPE-DM.

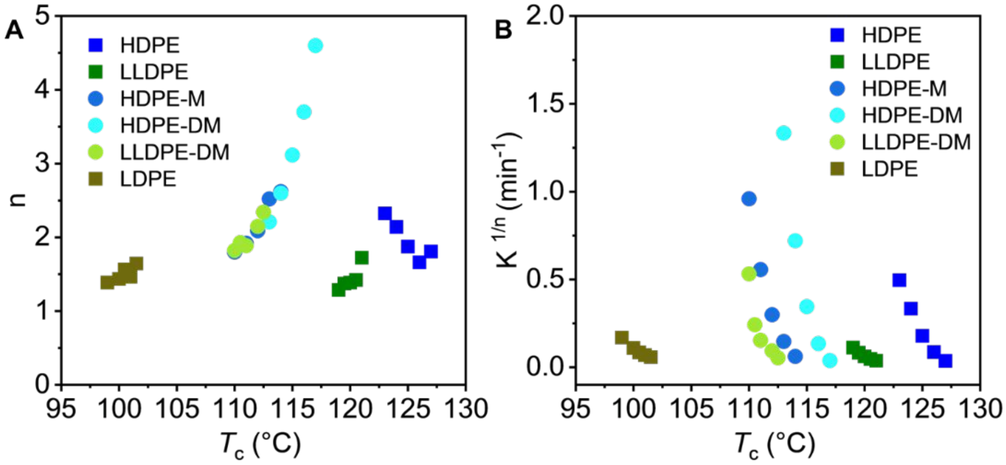

Figure S8. (A) Avrami exponent n and (B) Avrami constant K for the isothermal crystallization of HDPE, LLDPE, HDPE-M, HDPE-DM, LLDPE-DM and LDPE.

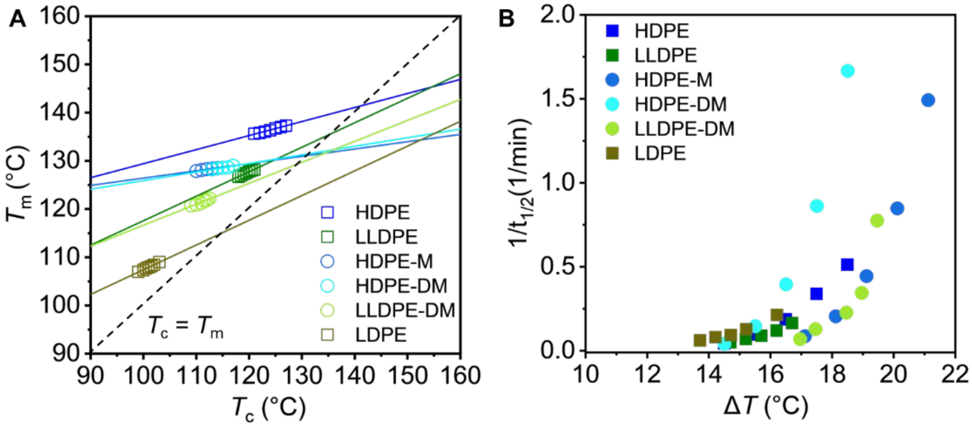

Figure S9 (A) Hoffmann−Weeks linear fit of melting temperatures (Tm) as a function of the crystallization temperature (Tc) and (B) overall crystallization rate as inverse of the half crystallization time ($1/t_{1/2}$) as a function of the supercooling ($\Delta T = T_m^0 - T_c$) for HDPE, LLDPE, HDPE-M, HDPE-DM, LLDPE-DM and LDPE.

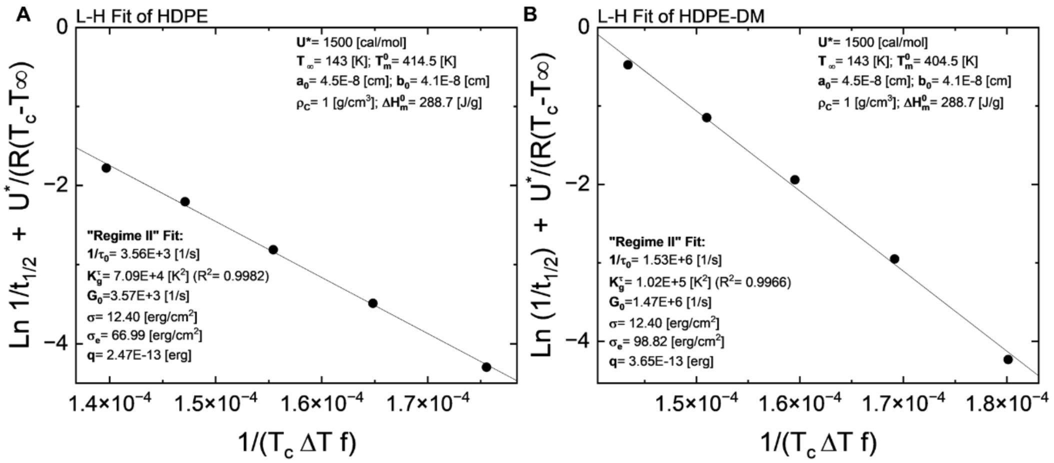

Figure S10. Lauritzen and Hoffman plot (A)HDPE and (B)HDPE-DM.

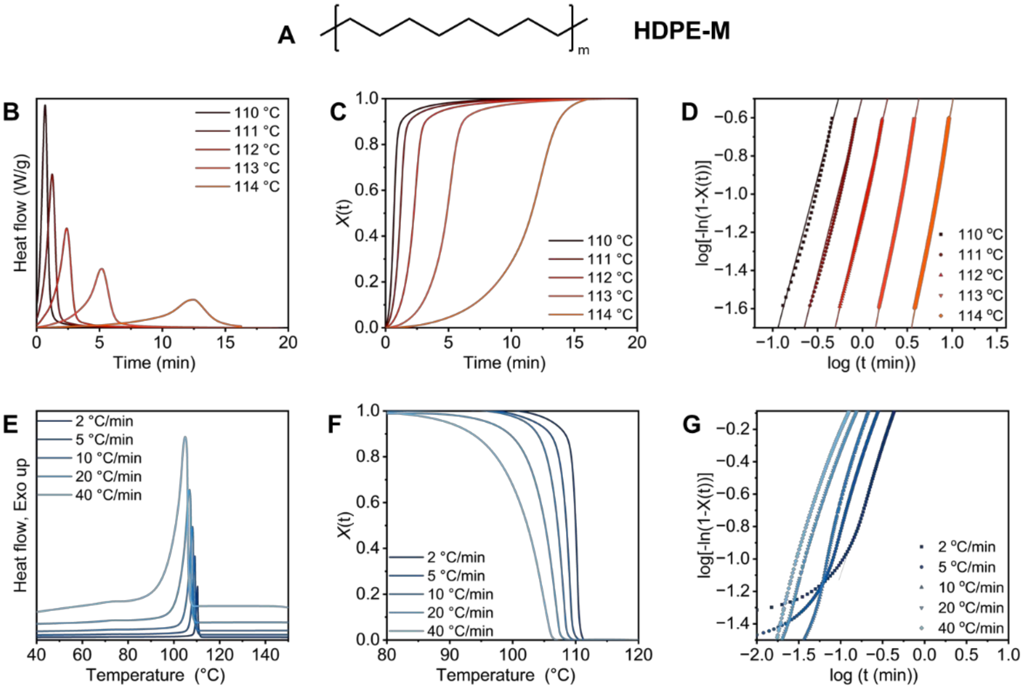

Figure S11. (A)Chemical structure, (B) representative isothermal crystallization exotherms at different crystallization temperatures, (C) relative crystallinity as a function of time for the isothermal crystallization, (D) linear fit of Avrami plot of isothermal crystallization in the range of Xt = 3% to 20% for HDPE-M, (E) representative non-isothermal crystallization exotherms at different cooling rates, (F) relative crystallinity as a function of temperature for the non-isothermal crystallization, and (G) Avrami plot in the range of Xt = 20% to 60% for the non-isothermal crystallization of HDPE-M

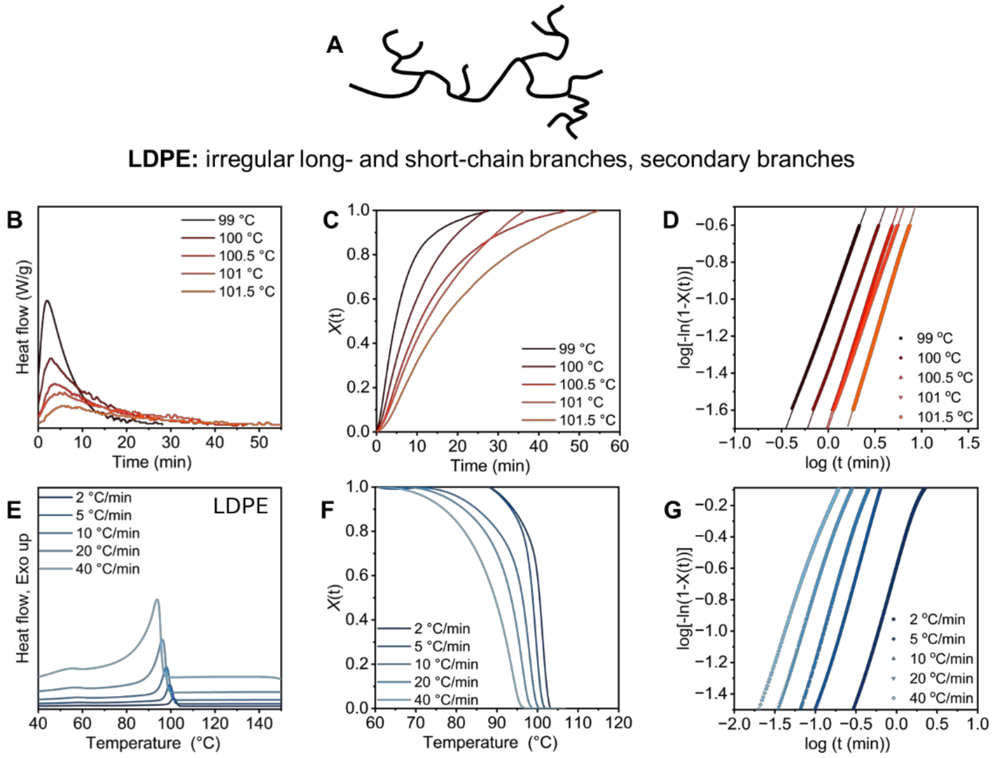

Figure S12. (A) Schematic illustration, (B) representative isothermal crystallization exotherms at different crystallization temperatures, (C) relative crystallinity as a function of time for the isothermal crystallization, (D) linear fit of Avrami plot of isothermal crystallization in the range of Xt = 3% to 20% for HDPE-M, (E) representative non-isothermal crystallization exotherms at different cooling rates, (F) relative crystallinity as a function of temperature for the non-isothermal crystallization, and (G) Avrami plot in the range of Xt = 20% to 60% for the non-isothermal crystallization of LDPE.

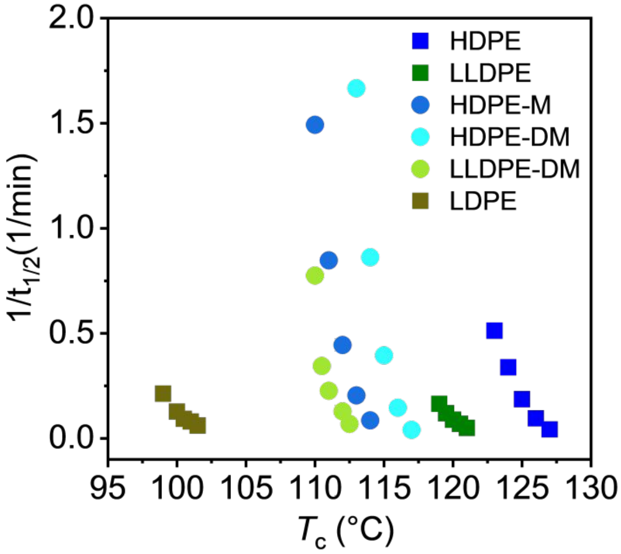

Figure S13. Overall crystallization rate as inverse of the half crystallization time ($1/t_{1/2}$) as a function of the crystallization temperature (T_c) for HDPE, LLDPE, HDPE-M, HDPE-DM, LLDPE-DM and LDPE.

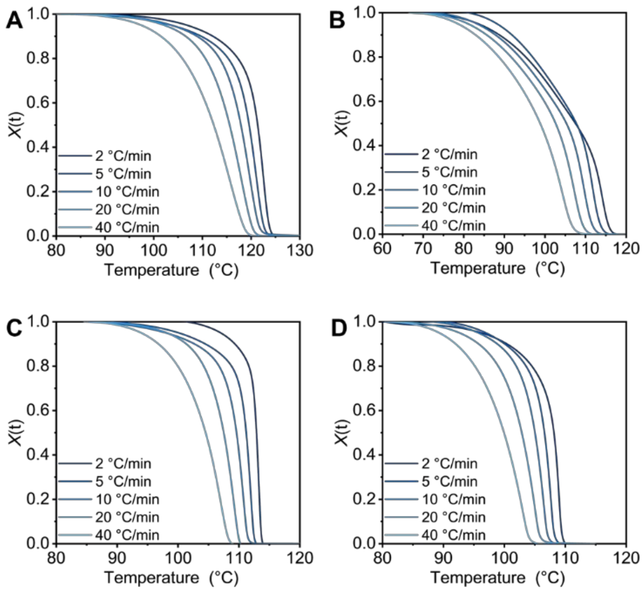

Figure S14. Relative crystallinity as a function of temperature for the non-isothermal crystallization of (A) HDPE, (B) LLDPE, (C) HDPE-M, (D) HDPE-DM and (E)LLDPE-DM.

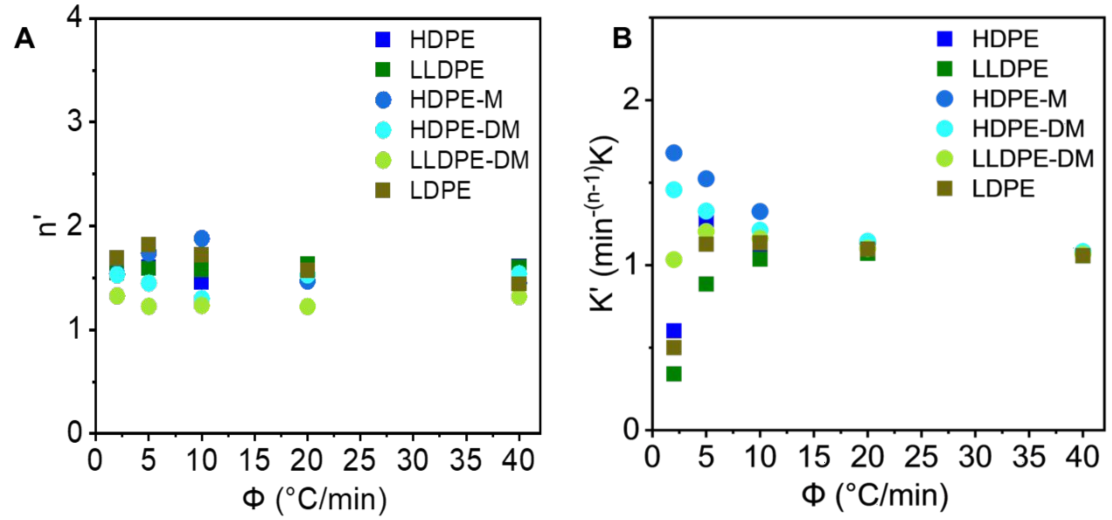

Figure S15. (A) Avrami exponent n' and (B) Jezirony-modified Avrami constant K' as a function of cooling rate for the non-isothermal crystallization of HDPE, LLDPE, HDPE-M, HDPE-DM, LLDPE-DM and LDPE.

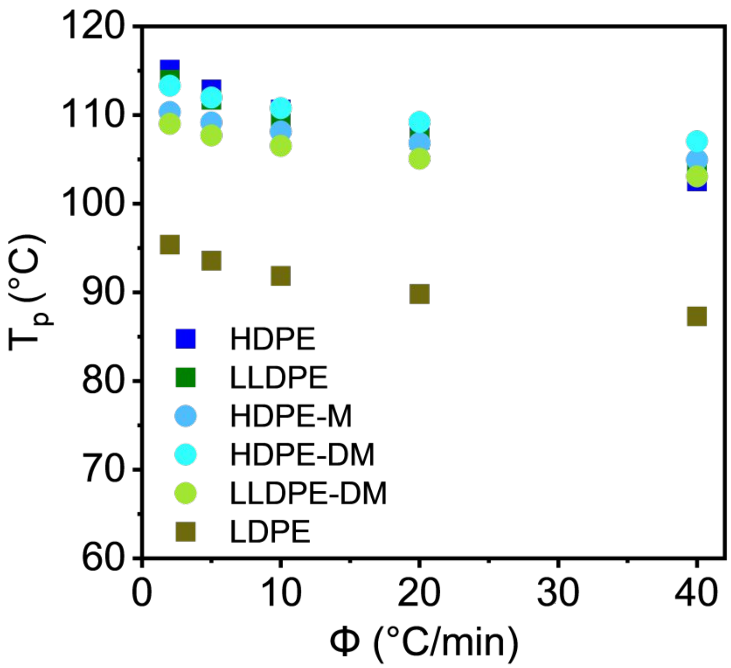

Figure S16. T_p of HDPE, LLDPE, HDPE-M, HDPE-DM, LLDPE-DM and LDPE. during non-isothermal crystallization as function of cooling rates.

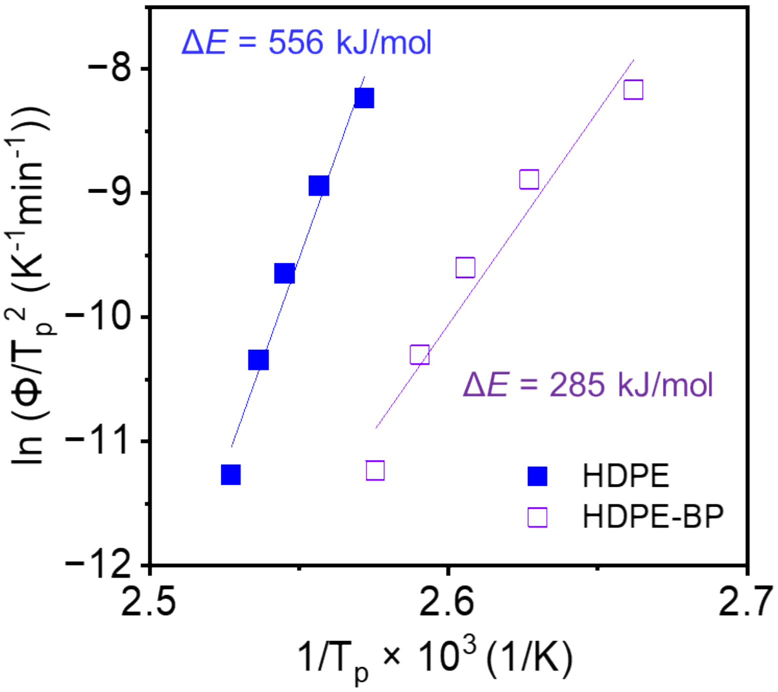

Figure S17. Kissinger plot for HDPE before precipitation (BP) and after precipitation.

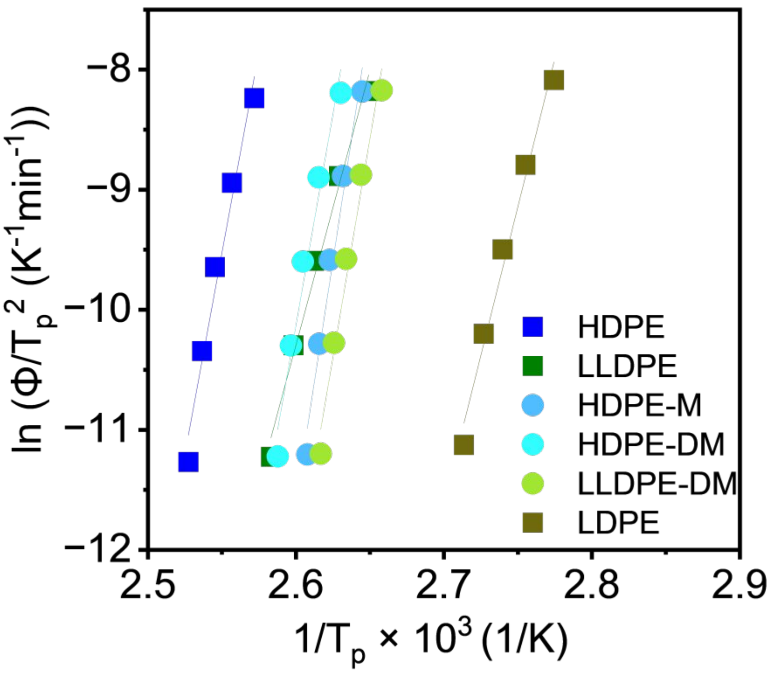

Figure S18. Kissinger plot for HDPE, LLDPE, HDPE-M, HDPE-DM, LLDPE-DM and LDPE.

Table S1. Values of T_m, X_c, T_m^0, ΔE, ester-to-methylene ratio (E:M), branch-to-methylene ratio (B:M), M_n and PDI.

	T_m (°C)	X_c (%)	T_m^0 (°C)	ΔE (kJ/mol)	E:M[a]	B:M[a]	M_w (Da)	PDI
HDPE	134	80.9	141.5	556	--	--	114k	9.9
LLDPE	121.5	34.4	135.7	380	--	--	105k	3.7
LDPE	105.4	31.5	115.2	411	--	--	--	--
HDPE-M	127.9	60	131.1	671	0	0	778k[a,b]	1.2[a,b]
HDPE-DM	128.7	51.2	131.5	584	1:413	0	509k[a,b]	1.5[a,b]
LLDPE-DM[c]	120.8	46.7	129.5	606	1:413	1:159	--	--

[a] Obtained from previous study [1]. [b] M_n and PDI were obtained from unsaturated polymers.[1] [c] The backbone molecular weight of LLDPE-DM is the same as HDPE-DM to isolate the effect of branching on crystallization behavior.

Table S2. Values of T_{onset}, T_p, and ΔT (=$T_m^0 - T_p$) at various cooling rates (ϕ) for all samples.

Sample	ϕ (°C /min)	T_{onset} (°C)	T_p (°C)	ΔT (°C)
HDPE	2	124.3	122.5	19.0
	5	123.3	121.1	20.4
	10	122.3	119.7	21.8
	20	121.2	117.9	23.6
	40	119.8	115.7	25.8
LLDPE	2	116.7	113.9	21.8
	5	114.7	111.6	24.1
	10	112.7	109.7	26.0
	20	110.5	108.2	27.5
	40	108.1	104.3	31.4
LDPE	2	98.1	95.4	19.8
	5	96.1	93.6	21.6

	10	94.3	91.8	23.4
	20	92.4	89.8	25.4
	40	90.3	87.3	27.9
HDPE-M	2	110.8	110.3	21.2
	5	109.8	109.2	22.3
	10	109.1	108.1	23.4
	20	107.9	106.8	24.7
	40	106.7	104.9	26.6
HDPE-DM	2	113.8	113.3	18.2
	5	112.7	111.9	19.6
	10	111.6	110.8	20.7
	20	110.4	109.2	22.3
	40	108.8	107.1	24.4
LLDPE-DM	2	109.6	109.0	20.5
	5	108.3	107.7	21.8
	10	107.3	106.5	23.0
	20	106.0	105.1	24.4
	40	104.4	103.1	26.4

(1) Li, X.; Suhail, A.; Mahadas, N. A.; Zhang, M.; Hu, Z.; Stefik, M.; Kuksenok, O.; Tang, C. Modulating Polyethylene Mimics with Degradability via Synthesis and Modeling. *Macromolecules* **2025**, *58* (4), 2094–2105. https://doi.org/10.1021/acs.macromol.5c00039.
